# Comparison of the teaching effect of problem-based learning and case-based learning teaching methods in dental endodontics education

**DOI:** 10.3389/fmed.2026.1800657

**Published:** 2026-05-12

**Authors:** Qun Li, Qiaojuan Zuo, Yanbo Xiao, Xun Liu, Yanan Yang, Bo Li

**Affiliations:** 1 Department of Stomatology, The First Hospital of Hunan University of Chinese Medicine, Changsha, Hunan, China; 2 Department of Radiology, The First Hospital of Hunan University of Chinese Medicine, Changsha, Hunan, China

**Keywords:** case-based learning (CBL), dental endodontics education, problem-based learning (PBL), teaching methods, undergraduate education

## Abstract

**Objective:**

This study aimed to compare the teaching effects of problem-based learning (PBL) and case-based learning (CBL) in dental endodontics education among 156 junior undergraduate dental students at the School of Stomatology, Hunan University of Chinese Medicine. The objective was to determine which method better promotes students' learning outcomes, including theoretical knowledge, practical skills, and learning attitudes.

**Methods:**

A total of 156 junior-year students were randomly divided into a PBL group and a CBL group, with 78 students in each. In the PBL group, students learned through problem-solving scenarios. Teachers presented real-life-like dental endodontics cases and guided students to explore relevant questions. Students in groups conducted independent research and discussion to find solutions. In the CBL group, real clinical cases were used. Teachers provided comprehensive case information, and students analyzed cases, discussed diagnosis and treatment plans in groups. After a semester of teaching intervention, both groups were evaluated by theoretical knowledge test, practical skill assessment, self-learning ability assessment, and satisfaction assessment of teaching model.

**Results:**

In the theoretical knowledge test, the PBL group showed a better understanding of in-depth concepts and knowledge integration, while the CBL group performed better in knowledge application related to case-specific situations. In practical skill assessment, the CBL group demonstrated more proficient operation skills. Regarding learning attitudes, students in the PBL group showed higher enthusiasm for independent exploration, while those in the CBL group had a stronger perception of the practical significance of learning. The satisfaction survey indicated that students in both groups were satisfied with their respective teaching methods, but for different reasons. The PBL group appreciated the cultivation of thinking ability, and the CBL group valued the connection with real-world practice.

**Conclusions:**

Both PBL and CBL teaching methods have positive impacts on dental endodontics education. PBL is more effective in cultivating students' independent learning, critical thinking, and knowledge integration abilities. CBL is better at helping students apply theoretical knowledge to clinical practice and enhancing their practical operation skills. Therefore, educators should choose teaching methods according to teaching objectives and students' characteristics, and even consider integrating the two methods to optimize the teaching effect in dental endodontics education.

## 1 Introduction

In the dynamic landscape of dental education, the pursuit of effective teaching methods is crucial for nurturing competent professionals (1, 2). For undergraduate dental students at the School of Stomatology, mastering the knowledge and skills of dental endodontics is crucial for their future clinical practice (3, 4). Dental endodontics, a specialized field within stomatology, focuses on diagnosing and treating diseases of the dental pulp and periapical tissues (5–7). Mastery of this discipline is essential as it forms the bedrock for students' future clinical practice, enabling them to provide high-quality care to patients.

However, traditional teaching approaches in dental endodontics often face limitations (8, 9). The conventional lecture-based model, where instructors dominate the teaching process by simply delivering knowledge, has proven to be less than ideal (10). Students are passive recipients of information, with limited opportunities to actively engage in the learning process (11). This passivity not only stifles students' creativity but also makes it difficult for them to bridge the gap between theoretical knowledge and real-world clinical applications (12). As a result, students may struggle to develop the critical thinking and problem-solving skills necessary for handling complex endodontic cases in their future careers (13).

In response to these challenges, innovative teaching methods such as problem-based learning (PBL) and case-based learning (CBL) have emerged. PBL is a student-centered teaching strategy that places emphasis on problem-solving (14). It starts with presenting students with real-life-like problems related to dental endodontics. For example, a problem could be formulating a treatment plan for a patient with a complex pulpitis case. Students are then encouraged to take the initiative in seeking knowledge, conducting research, and collaborating with their peers to find solutions. Through this process, PBL aims to enhance students' self-learning abilities, critical thinking, and their capacity to analyze and solve problems independently (15).

CBL, on the other hand, centers around real clinical cases. It immerses students in actual patient scenarios, providing them with a more realistic learning environment (16). By analyzing and discussing these cases, students can better understand how to apply theoretical knowledge in a clinical setting (17). For instance, in a CBL session, students might analyze a case of apical periodontitis, considering factors like the patient's medical history, radiographic findings, and symptoms to determine the most appropriate treatment approach. This method helps students develop practical skills, improve their clinical judgment, and gain a deeper understanding of the decision-making processes involved in dental endodontics (18).

While numerous studies have explored the application of PBL and CBL in medical and dental education, there is a scarcity of research specifically comparing their effectiveness in dental endodontics education for students at the School of Stomatology. Understanding which approach better suits the unique learning needs of these students, or how these methods can be optimized in the context of the local curriculum, is of great significance.

This study aims to fill this research gap by comparing the teaching effects of PBL and CBL in dental endodontics education among 156 junior undergraduate dental students at the School of Stomatology, Hunan University of Chinese Medicine. By evaluating students' learning outcomes, including theoretical knowledge, practical skills, self-learning ability assessment, and satisfaction with the teaching mode, this research endeavors to provide educators with evidence-based insights. These insights can guide the selection and implementation of teaching methods, ultimately enhancing the quality of dental endodontics education and better preparing students for the challenges of their future clinical practice.

## 2 Materials and methods

### 2.1 Study participants

One hundred and fifty-six junior undergraduate dental students from the School of Stomatology, Hunan University of Chinese Medicine were selected as the subjects of this study. Prior to recruitment, a power analysis using G^*^Power 3.1.9.7 indicated that a total sample size of 128 (64 per group) would provide a power (1–β) of 0.80 at an α level of 0.05 with a medium effect size (Cohen's *d* = 0.50). Thus, our final sample of 156 students ensured sufficient statistical power (>0.85) to detect significant differences. These students were in their third year of study, at a crucial stage in their dental education where they were about to delve deeper into the specialized knowledge of dental endodontics. Before the study, all students had similar pre-course academic performances in basic dental courses, as evidenced by their previous semester's grades in courses such as Oral anatomy and physiology and Oral biology.

**Figure 1:**
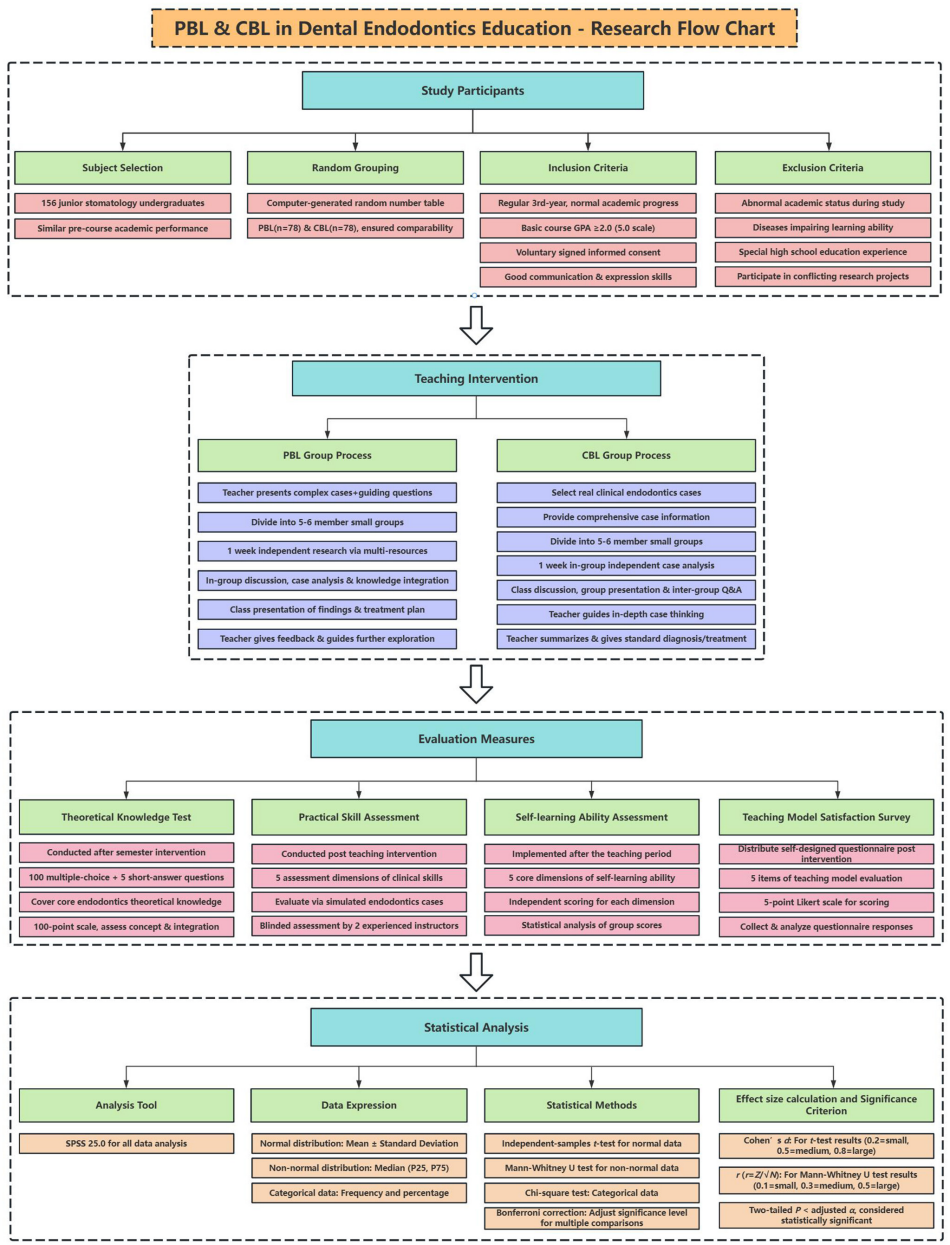
Overview of the study design.

Using a computer-generated random number table, the 156 students were randomly assigned into two groups: the PBL group and the CBL group, with 78 students in each. This randomization process aimed to ensure that the two groups were comparable in terms of students' initial knowledge levels, learning abilities, and academic backgrounds, minimizing potential confounding factors that could affect the teaching outcomes. Inclusion criteria: (1) Be undergraduate students majoring in Stomatology at Hunan University of Chinese Medicine, with a normal academic progress in the third year of undergraduate study. (2) Have a comprehensive average GPA of 2.0 or above (on a 5.0 scale) in previous basic stomatology courses, and show no significant difference in these scores between the two groups (*P* > 0.05). (3) Voluntarily sign the informed consent form to participate in the study. (4) Have good reading, writing and oral expression abilities, with good communication skills in group learning activities. Exclusion criteria: (1) Students with abnormal academic situations such as suspension, transfer, or repetition during the research period. (2) Students suffering from diseases that seriously affect cognitive function, mental illnesses, or physical diseases that significantly impact learning ability. (3) Students with special education experiences, like individualized one-on-one tutoring or long-term absences in high school. (4) Students participating in other educational research projects that may interfere with the study of endodontics or the evaluation of teaching methods in this study (Figure 1).

### 2.2 Teaching intervention

To eliminate potential bias, both groups strictly followed the same syllabus, core knowledge points, and total class hours, with materials standardized by a senior instructor committee to ensure equivalent difficulty and learning objectives.

#### 2.2.1 PBL group

For the PBL group, the teaching process was centered around well-designed problem-scenarios. In each PBL session, teachers first presented a complex dental endodontics case, for example, a case of a patient with a long-standing history of toothache, accompanied by swelling and discomfort. Along with the case, a series of guiding questions were provided. These questions were designed to gradually lead students to think about the diagnosis and treatment of the case, such as “What could be the possible causes of this patient's symptoms considering the long-standing nature of the toothache?”, “How would you use different diagnostic tools to confirm the diagnosis?”, and “What are the potential treatment plans and their associated risks and benefits?”.

Students were organized into small groups of 5–6 members. Each group was given 1 week to conduct independent research. They were encouraged to utilize various resources, including textbooks, academic databases like PubMed and China National Knowledge Infrastructure (CNKI), and dental professional websites. During this period, students were required to actively discuss within the group, sharing their findings and ideas. They needed to analyze the case from multiple perspectives, integrating knowledge from different dental and related medical fields.

After the research and group discussion, each group was required to present their findings and proposed solutions in a class presentation. The presentation included a detailed analysis of the case, the reasoning behind their diagnosis, and the proposed treatment plan. Teachers then provided comprehensive feedback, guiding students to further refine their understanding, correct any misunderstandings, and explore alternative approaches.

#### 2.2.2 CBL group

In the CBL group, teaching was based on real-world clinical cases. Teachers selected a series of diverse and representative cases from the hospital's dental endodontics department. Each case was presented with comprehensive information, including the patient's medical history, detailed clinical symptoms, radiographic images (such as X-rays and cone-beam computed tomography scans), and previous treatment attempts if any.

Similar to the PBL group, students in the CBL group were divided into small groups of 5–6. They were given 1 week to analyze the case independently within the group. The analysis required students to consider all aspects of the case, from formulating a diagnosis to developing a comprehensive treatment plan. They needed to take into account the patient's overall health condition, the specific characteristics of the dental lesion, and the available treatment options.

After the group analysis, a class discussion was held. Each group presented their diagnosis and treatment plan, and other groups were encouraged to ask questions and provide suggestions. Teachers played a guiding role during the discussion, helping students to think more deeply about the case, such as exploring the reasons for different treatment options, potential complications, and how to deal with unexpected situations during treatment. Finally, teachers summarized the key points of the case, compared different groups' solutions, and provided the standard diagnosis and treatment plan based on current clinical guidelines.

### 2.3 Evaluation measures

#### 2.3.1 Theoretical knowledge test

At the end of the semester-long teaching intervention, a comprehensive theoretical knowledge test (additional file 1) was administered to both groups. The test consisted of 100 multiple-choice questions and five short-answer questions, covering a wide range of topics in dental endodontics, including the anatomy and physiology of the dental pulp and periapical tissues, disease etiology and pathogenesis, diagnostic methods, and treatment techniques. The multiple-choice questions were designed to assess students' understanding of basic concepts, while the short-answer questions aimed to evaluate their ability to integrate knowledge and provide in-depth analysis. The test was graded on a 100-point scale, with higher scores indicating a better understanding of the theoretical knowledge.

#### 2.3.2 Practical skill assessment

Upon completion of the teaching intervention, a practical skill assessment (additional file 2) was conducted for students in both the PBL and CBL groups. This assessment was structured to evaluate their practical proficiency in dental endodontics. It covered multiple aspects, including instrument operation, such as the proper use of dental files during root canal preparation. Therapeutic technical proficiency was gauged through tasks like root canal treatment. Material selection rationality was tested based on students' ability to choose appropriate substances for different cases. Aseptic operation compliance was strictly monitored, and the accuracy of clinical judgment was evaluated using simulated cases. Each component was scored on a set scale, and the total scores of the two groups were statistically analyzed to determine the impact of PBL and CBL on practical skill development. Two experienced dental endodontics instructors, who were blinded to the group assignments of the students, conducted the assessments to ensure objectivity.

#### 2.3.3 Self-learning ability assessment

Following the teaching period, a comprehensive self-learning ability assessment (additional file 3) was administered to students in the PBL and CBL groups. The assessment centered around five key dimensions. First, learning plan and management ability was evaluated by looking at how students organized study time and set goals. Second, information acquisition and screening ability was measured through their use of various resources. Third, knowledge understanding and transformation ability was tested based on applying learned knowledge to real-world scenarios. Fourth, problem-solving and reflection ability was examined using endodontic problems. Finally, learning method innovation and application ability was considered. Each dimension was scored, and statistical analysis of the group scores aimed to identify which teaching method was more effective in fostering self-learning skills crucial for students' long-term growth.

#### 2.3.4 Satisfaction assessment of teaching model

A self-designed satisfaction assessment of teaching model questionnaire (additional file 4) was distributed to students in both groups at the end of the teaching intervention. The questionnaire included five items covering aspects such as effectiveness of teaching methods, learning effect improvement, curriculum rationality, faculty guidance role, and learning to experience feelings. Students were asked to rate their responses on a 5-point Likert scale, where 1 represented “strongly disagree” and 5 represented “strongly agree”. The responses were collected and analyzed to understand students' attitudes toward the PBL and CBL teaching methods.

### 2.4 Statistical analysis

Statistical analyses were performed using IBM SPSS Statistics 25.0 (IBM Corp., Armonk, NY, USA). Categorical data are presented as frequencies and percentages (*n*, %) and were compared between groups using the Chi-square (χ^2^) test. Continuous data are expressed as mean ± standard deviation (± *s*) or median (interquartile range [IQR]) depending on their distribution. The normality of continuous data was assessed using the Shapiro-Wilk test, and the homogeneity of variance was evaluated using Levene's test. For comparisons between the PBL and CBL groups, independent-samples *t*-tests were employed for normally distributed continuous variables with equal variances (e.g., total scores of theoretical knowledge and practical skills). For non-normally distributed data or ordinal variables (e.g., specific sub-item scores and satisfaction ratings), the non-parametric Mann–Whitney *U*-test was used. To control the inflated Type I error rate arising from multiple comparisons across various dimensions, the Bonferroni correction was applied to adjust the significance threshold (α). Furthermore, effect sizes were reported to assess the magnitude of differences: Cohen's *d* was used for *t*-tests (0.2 = small, 0.5 = medium, 0.8 = large), and *r* (*r* = *Z* / √*N*}) was used for Mann–Whitney *U*-tests (0.1 = small, 0.3 = medium, 0.5 = large). A two-tailed *P*-value of less than the adjusted significance level was considered statistically significant.

## 3 Results

### 3.1 Baseline characteristics

**Figure 2:**
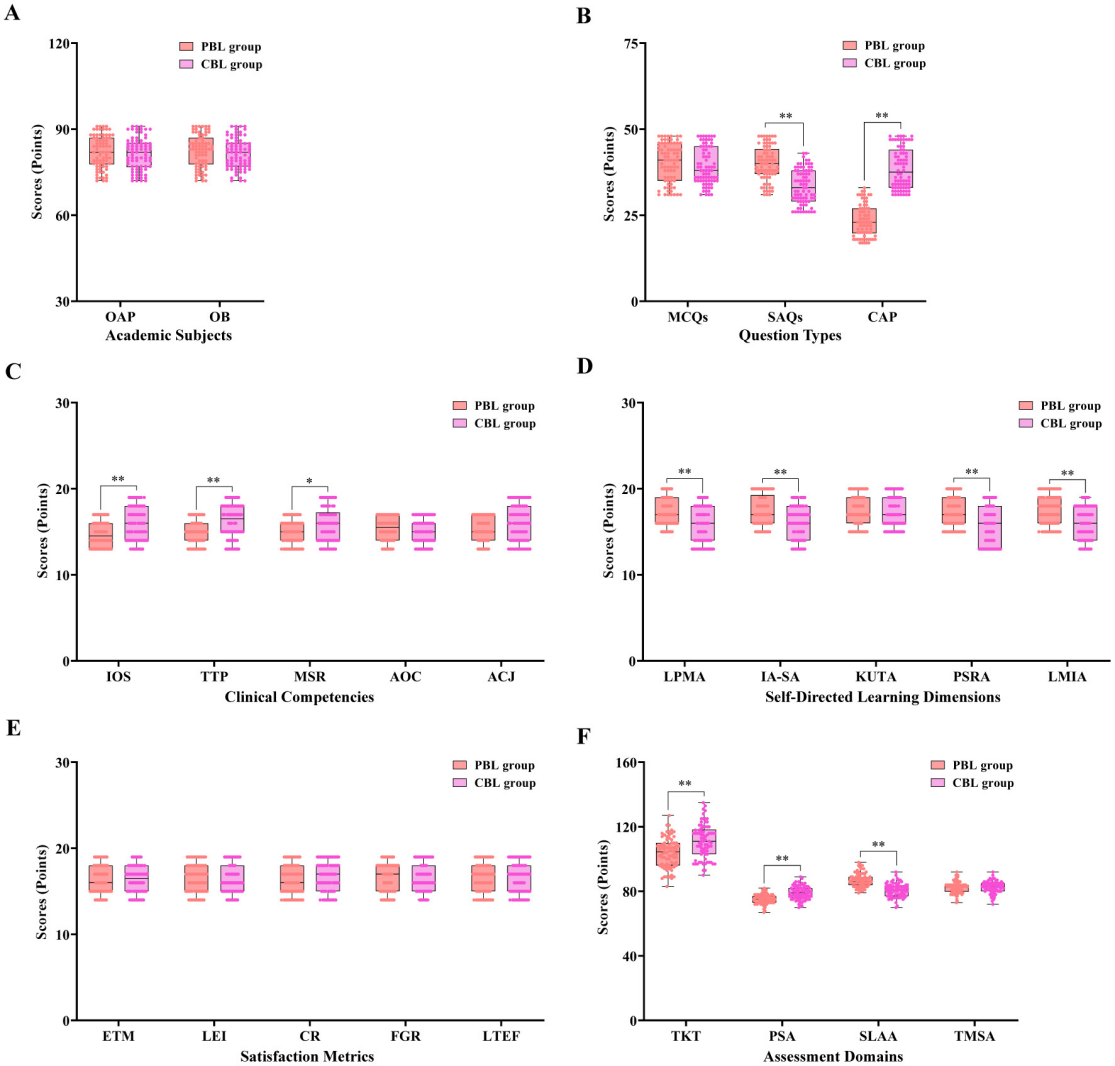
Comparative analysis of teaching effects between PBL and CBL groups in endodontics education. This figure illustrates the comprehensive comparison of teaching effects between the problem-based learning (PBL) group and case-based learning (CBL) group in dental endodontics education. Box-and-whisker plots with overlaid individual data points were employed to visualize the distribution of assessment scores across multiple dimensions: **(A)** Baseline knowledge scores in oral anatomy and physiology (OAP) and oral biology (OB). **(B)** Theoretical knowledge test scores, including multiple-choice questions (MCQs), short-answer questions (SAQs), and case-application problems (CAP). **(C)** Practical skill assessment scores, covering instrument operation specification (IOS), therapeutic technical proficiency (TTP), material selection rationality (MSR), aseptic operation compliance (AOC), and accuracy of clinical judgment (ACJ). **(D)** Self-learning ability assessment scores, including learning plan and management ability (LPMA), information acquisition and screening ability (IA-SA), knowledge understanding and transformation ability (KUTA), problem solving and reflection ability (PSRA), and learning method innovation and application ability (LMIA). **(E)** Teaching satisfaction assessment scores, including effectiveness of teaching methods (ETM), learning effect improvement (LEI), curriculum rationality (CR), faculty guidance role (FGR), and learning experience feelings (LTEF). **(F)** Total scores of four core outcome dimensions: theoretical knowledge test (TKT), practical skill assessment (PSA), self-learning ability assessment (SLAA), and teaching model satisfaction assessment (TMSA). Independent-samples *t*-test or Mann–Whitney *U*-test was used for group comparisons, depending on data distribution. *P < 0.05, **P < 0.01 denote statistically significant differences between the PBL and CBL groups.

Prior to the initiation of the teaching intervention, a comprehensive assessment of the baseline characteristics of the 156 junior undergraduate dental students from the School of Stomatology, Hunan University of Chinese Medicine was conducted. In terms of demographic data, the distribution of gender across the two groups was similar. A chi-square test confirmed that this difference was not statistically significant (χ^2^=0.245, *P* > 0.05). Regarding age, a Mann-Whitney *U*-test showed no significant difference between the two groups (*Z* = −0.196, *P* > 0.05). This indicates that the students in both groups were at a similar stage of cognitive and academic development, which is crucial for a fair comparison of the teaching methods. Academic performance in pre-course basic dental courses was a key factor in ensuring group comparability. As indicated by their previous semester's grades in courses like Oral anatomy and physiology and Oral biology, the two groups had comparable academic achievements. Mann–Whitney *U*-test for Oral anatomy and physiology course showed no significant difference (*Z*_*Oralanatomyandphysiology*_=-0.900, *P* > 0.05), and independent samples *t*-test for Oral biology course showed no significant difference (*t*_*Oralbiology*_=0.912, *P* > 0.05). In conclusion, the randomization process effectively created two groups of students with comparable baseline characteristics in terms of gender, age, academic performance in relevant pre-course subjects. This provides a solid foundation for accurately evaluating and comparing the teaching effects of PBL and CBL in dental endodontics education, ensuring that the observed differences in learning outcomes can be reasonably attributed to the distinct teaching methodologies rather than pre-existing disparities among the students (Table 1, Figure 2A).

**Table 1:** Baseline demographic characteristics of students in the PBL and CBL groups.

Item	PBL group (*n* = 78)	CBL group (*n* = 78)	*χ^2^/t/Z*	*P*
**Gender**			*χ^2^*= 0.245	0.620
Male	28	31		
Female	50	47		
Age	21 (20, 22)	21 (20, 22)	*Z*=-0.196	0.845
**Basic dental course scores**				
Oral anatomy and physiology	82 (77.75, 87.00)	82 (76.75, 85.25)	*Z*=-0.900	0.368
Oral biology	82.360 ± 5.622	81.560 ± 5.258	*t* = 0.912	0.363

Baseline data showed no significant differences between groups (*P* > 0.05).

### 3.2 Theoretical knowledge test

The comparative results of the theoretical knowledge assessment between the PBL and CBL groups are detailed in Table 2. Prior to the core analysis, the normality of the data distribution was rigorously evaluated using the Shapiro-Wilk test. The results indicated that the total theoretical scores for both the PBL group and the CBL group followed a normal distribution (*P* > 0.05). Additionally, Levene's test confirmed the homogeneity of variance between the two groups (*P* > 0.05), thereby justifying the application of the independent-samples *t*-test for the comparison of total scores. The CBL group achieved a significantly higher total score than the PBL group. To assess the educational significance of this finding, Cohen's *d* was calculated, yielding a value of 0.74, which represents a medium-to-large effect size (*P* < 0.001). This suggests that the CBL approach has a substantial impact on the overall acquisition of theoretical knowledge in endodontics. Regarding the sub-item analysis, the scores for multiple-choice questions, short-answer questions, and case-application questions exhibited non-normal distributions; consequently, the non-parametric Mann–Whitney *U*-test was employed. In the multiple-choice section, no statistically significant difference was found between the two groups (*P* = 0.224), with a negligible effect size (*r* = 0.10). However, the PBL group demonstrated a superior performance in short-answer questions, which primarily evaluate the integration and synthesis of theoretical concepts. The difference was statistically significant with a large effect size (*r* = 0.54, *P* < 0.001). Conversely, the CBL group significantly outperformed the PBL group in case-application questions, which measure the ability to apply theoretical knowledge to clinical scenarios (*P* < 0.001). The calculated effect size for this dimension was exceptionally large (*r* = 0.84), underscoring the decisive advantage of CBL in clinical reasoning. To address the risk of Type I errors arising from multiple comparisons across these dimensions, the Bonferroni correction was applied, adjusting the significance threshold to α' = 0.0125. Under this more conservative criterion, the observed differences in total scores, short-answer questions, and case-application questions remained statistically significant (Figures 2B, F).

**Table 2:** Comparison of theoretical knowledge test scores and effect sizes between the PBL and CBL groups.

Item	PBL group (*n* = 78)	CBL group (*n* = 78)	*t/Z*	*P*	Effect size
Multiple-choice questions scores	41 (35.00, 46.00)	38 (35.00, 45.00)	*Z*=-1.217	0.224	*r* = 0.10
Short-answer questions scores	40 (37.44, 25.00)	33 (29.00, 38.00)	*Z*=-6.787	<0.001^**^	*r* = 0.54
Case-application problem scores	23 (19.75, 27.00)	37.5 (33.00, 44.00)	*Z*=-10.582	<0.001^**^	*r* = 0.84
Total scores	103.870 ± 9.226	111.190 ± 10.439	*t*=-4.641	<0.001^**^	*d* = 0.74

Effect sizes are reported as Cohen's d or r (r = Z / √N), and

^**^P < 0.01 denotes significance after Bonferroni correction.

### 3.3 Practical skill assessment

The comparative analysis of practical skill performance between the PBL and CBL groups is presented in Table 3. To ensure the appropriateness of the statistical methods, the normality of the total practical scores was first assessed using the Shapiro-Wilk test, which confirmed a normal distribution for both groups (*P* > 0.05). Consequently, an independent-samples *t*-test was conducted for the total score comparison. The CBL group demonstrated a markedly superior performance with a total score of 79.00 ± 4.13, compared to 75.23 ± 2.98 in the PBL group (*t* = −6.535, *P* < 0.001). The calculated Cohen's *d* for the total score was 1.05, indicating a large effect size and suggesting that the CBL model provides a substantial advantage in enhancing overall clinical operative proficiency in endodontics. For the specific sub-item assessments, including instrument operation, technical proficiency, material selection, aseptic compliance, and clinical judgment, the data exhibited non-normal distributions and were thus analyzed using the Mann–Whitney *U*-test. The CBL group achieved significantly higher scores in instrument operation (*P* < 0.001, *r* = 0.29) and technical proficiency (*P* < 0.001, *r* = 0.33). In the dimension of material selection, the CBL group also maintained a statistically significant lead (*P* = 0.007, *r* = 0.21). However, no significant difference was observed in aseptic compliance (*P* = 0.319). Notably, regarding clinical judgment, although the CBL group scores were numerically higher (*P* = 0.040), this difference was not considered statistically significant after the application of the Bonferroni correction. To control for Type I error across the five sub-items, the significance threshold was adjusted to α' = 0.01. Under this conservative criterion, the *P*-value of 0.040 for clinical judgment exceeded the threshold, indicating that while a positive trend exists, further study may be required to confirm a definitive advantage. Overall, the significant improvements in total scores and core technical dimensions with large effect sizes underscore the efficacy of CBL in bridging the gap between theoretical knowledge and clinical practice (Figures 2C, F).

**Table 3:** Comparison of practical skill assessment scores and effect sizes between the PBL and CBL groups.

Item	PBL group (*n* = 78)	CBL group (*n* = 78)	*t/Z*	*P*	Effect size
Instrument operation specification	14.50 (13.00, 16.00)	16.00 (14.00, 18.00)	*Z*=-3.644	<0.001^**^	*r* = 0.29
Therapeutic technical proficiency	15.00 (14.00, 16.00)	16.50 (15.00, 18.00)	*Z*=-4.151	<0.001^**^	*r* = 0.33
Material selection rationality	15.00 (14.00, 16.00)	16.00 (14.00, 17.25)	*Z*=-2.690	0.007^*^	*r* = 0.21
Aseptic operation compliance	15.50 (14.00, 17.00)	15.00 (14.00, 16.00)	*Z*=-0.996	0.319	*r* = 0.08
Accuracy of clinical judgment	15.00 (14.00, 17.00)	16.00 (14.00, 18.00)	*Z*=-2.053	0.040	*r* = 0.16
Total score	75.230 ± 2.976	79.00 ± 4.134	*t*=-6.535	<0.001^**^	*d* = 1.05

Data are presented as ± s or median (IQR) with corresponding Cohen's d or r effect sizes; significance levels are

^*^P < 0.05 and

^**^P < 0.01 following Bonferroni correction, noting that “Clinical Judgment” (*P* = 0.040) is not marked with an asterisk as it exceeds the adjusted threshold of α' = 0.01 (0.05/5 dimensions).

### 3.4 Self-learning ability assessment

The comparative results regarding students' self-learning ability after the teaching intervention are summarized in Table 4. To ensure a rigorous evaluation of the five distinct dimensions of self-learning, the data were first subjected to the Shapiro-Wilk test, which revealed a non-normal distribution across all categories. Consequently, the non-parametric Mann–Whitney *U*-test was employed for group comparisons. Furthermore, to mitigate the risk of Type I errors arising from multiple comparisons across these five dimensions, the Bonferroni correction was applied, adjusting the significance threshold to α' = 0.01 (0.05/5). The results indicated that the PBL group achieved a significantly higher total score in self-learning ability compared to the CBL group (*Z* = −7.643, *P* < 0.001). This difference was characterized by a large effect size (*r* = 0.61), underscoring the substantial impact of the PBL model on fostering independent learning habits. Specifically, the PBL group demonstrated statistically significant superiority in four out of five dimensions: “Plan and Management” (*r* = 0.34, *P* < 0.001), “Information Acquisition” (*r* = 0.31, *P* < 0.001), “Problem Solving” (*r* = 0.33, *P* < 0.001), and “Method Innovation” (*r* = 0.37, *P* < 0.001). In each of these categories, the adjusted *P*-values remained well below the conservative threshold of 0.01. In contrast, no statistically significant difference was observed between the two groups in the “Knowledge Transformation” dimension (*P* = 0.376), with a negligible effect size (*r* = 0.07). This suggests that while both models are effective in helping students process and internalize dental information, the PBL approach—by requiring students to navigate ill-structured problems and lead their own research—is markedly more effective in enhancing the strategic and innovative components of autonomous learning. The robust effect sizes and the persistence of significance after Bonferroni adjustment provide strong evidence for the role of PBL in developing the self-directed learning competencies essential for future dental practice (Figures 2D, F).

**Table 4:** Comparison of self-learning ability assessment scores and effect sizes between the PBL and CBL groups.

Item	PBL group (*n* = 78)	CBL group (*n* = 78)	*Z*	*P*	^EffectSize(r)^
Learning plan and management ability	17.00 (16.00, 19.00)	16.00 (14.00, 18.00)	−4.270	<0.001^**^	0.34
Information acquisition and screening ability	17.00 (16.00, 19.25)	16.00 (14.00, 18.00)	−3.849	<0.001^**^	0.31
Knowledge understanding and transformation ability	17.00 (16.00, 19.00)	17.00 (16.00, 19.00)	−0.886	0.376	0.07
Problem solving and reflection ability	17.00 (16.00, 19.00)	16.00 (13.00, 18.00)	−4.156	<0.001^**^	0.33
Learning method innovation and application ability	18.00 (16.00, 19.00)	16.00 (14.00, 18.00)	−4.669	<0.001^**^	0.37
Total score	86.00 (83.75, 89.00)	81.00 (77.00, 84.00)	−7.643	<0.001^**^	0.61

All dimensions are expressed as median (IQR) and compared via Mann–Whitney U-test with effect size r reported;

^**^P < 0.01 indicates statistical significance after Bonferroni correction (α' = 0.05/5 = 0.01) to control for Type I error across multiple dimensions.

### 3.5 Satisfaction assessment of teaching model

The satisfaction assessment of the teaching model indicated that students in both the PBL and CBL groups were generally satisfied with their respective teaching methods, but for different reasons. In the effectiveness of teaching methods aspect, the PBL group highly valued the cultivation of thinking abilities. They appreciated how the problem-solving scenarios encouraged them to think critically, analyze complex situations, and develop independent solutions. Many PBL students reported feeling more confident in their ability to approach novel endodontic problems after the PBL teaching process. The CBL group, on the other hand, highly regarded the connection with real-world practice. They believed that analyzing real clinical cases was extremely beneficial for their future careers. CBL students felt that this teaching method made the learning content more tangible and relevant, as they could directly apply what they learned to real patient situations. Regarding learning effect improvement, both groups showed high levels of agreement, indicating that both PBL and CBL were effective in enhancing students' knowledge and skills in dental endodontics. In terms of curriculum rationality, the PBL group appreciated the logical structure of the problem-solving scenarios, which gradually led them to explore deeper concepts. The CBL group, however, thought the curriculum was rational because it was based on real-life cases, providing a practical framework for learning. The faculty guidance role was also valued by both groups. PBL students found teachers' feedback during the problem-solving process helpful in refining their understanding, while CBL students appreciated teachers' guidance in case analysis and decision-making. When it came to learning experience feelings, the PBL group enjoyed the sense of achievement from solving complex problems, and the CBL group liked the real-world exposure and the feeling of being part of a clinical team. These findings, supported by negligible effect sizes (all *r* < 0.15), indicate that both pedagogical approaches were equally well-received by the students. The total score for satisfaction was high in both groups, but the reasons for their satisfaction clearly reflected the unique characteristics of the PBL and CBL teaching methods (Table 5, Figures 2E, F).

**Table 5:** Comparison of satisfaction ratings and effect sizes for teaching models between the PBL and CBL groups.

Item	PBL group (*n* = 78)	CBL group (*n* = 78)	*Z*	*P*	Effect Size (*r*)
Effectiveness of teaching methods	16.00 (15.00, 18.00)	16.50 (15.00, 18.00)	−0.121	0.904	0.01
Learning effect improvement	17.00 (15.00, 18.00)	16.00 (15.00, 18.00)	−0.837	0.403	0.07
Curriculum rationality	16.00 (15.00, 18.00)	17.00 (15.00, 18.00)	−1.521	0.128	0.12
Faculty guidance role	17.00 (15.00, 18.00)	16.00 (15.00, 18.00)	−0.074	0.941	0.01
Learning to experience feelings	17.00 (15.00, 18.00)	17.00 (15.00, 18.00)	−0.115	0.908	0.01
Total score	83.00 (79.75, 84.00)	83.00 (80.00, 85.00)	−0.855	0.393	0.07

Satisfaction ratings are presented as median (IQR) and analyzed using the Mann–Whitney U-test with effect size r; no statistically significant differences were observed after Bonferroni correction (*P* > 0.01), indicating equivalent high satisfaction with both teaching models.

## 4 Discussion

Compared with traditional lecture-based learning (LBL), active models like PBL and CBL more effectively bridge the gap between theory and clinical practice. Recent studies confirm that while LBL is efficient for knowledge transmission, PBL and CBL significantly enhance deep learning and clinical reasoning in dental education (19, 20). These methods are particularly vital for endodontics, which demands high levels of spatial logic and clinical judgment. The results of this study indicate that both PBL and CBL teaching methods have their own unique advantages in dental endodontics education for junior undergraduate students at the School of Stomatology, Hunan University of Chinese Medicine. These findings not only confirm the effectiveness of these innovative teaching methods but also provide valuable insights into how to optimize dental education. By juxtaposing these two pedagogical approaches, we can better understand their respective strengths and limitations, guiding educators toward more informed teaching decisions.

In the realm of theoretical knowledge acquisition, the PBL group demonstrated a superior ability in understanding in-depth concepts and knowledge integration (21, 22). This superiority can be explained by the Constructivist Learning Theory, which posits that learners actively build their own knowledge through experience and interaction rather than passively receiving information. The PBL framework, which mandates students to independently research and analyze problems, functions as “scaffolding,” allowing students to synthesize knowledge from various dental and related medical disciplines into a coherent personal framework. Recent studies have similarly highlighted that such integrative pedagogical approaches are vital for advancing interdisciplinary learning in medical fields (19). Conversely, the CBL group's proficiency in case-application problems and practical skills underscores the value of Situated Learning Theory. Situated learning emphasizes that mastery is best achieved within the context and culture in which it is applied (20–23). This is particularly critical in dental endodontics, a discipline characterized by its heavy reliance on imaging (such as X-rays and CBCT scans) and high-precision mechanical operations (like root canal preparation). By immersing students in realistic clinical scenarios early on, CBL reduces the “cognitive load” during the transition from classroom to clinic, resulting in more accurate clinical judgments and proficient therapeutic techniques. This alignment between teaching methodology and professional practice is consistent with recent findings regarding technology-enhanced and case-integrated learning in dental education.

Self-learning ability is a cornerstone of a student's long-term academic and professional growth (24). The PBL group's stronger performance in learning planning, information acquisition, and problem-solving indicates that PBL is highly effective in fostering independent learning. These skills are essential for lifelong learning in the rapidly evolving field of dentistry. While CBL also promotes self-learning through practice-oriented engagement, the proactive nature of PBL specifically targets the development of critical thinking and knowledge transformation. This is supported by contemporary research suggesting that novel teaching strategies like PBL significantly enhance problem-solving and critical thinking abilities for healthcare students (25). The satisfaction assessment revealed that both methods are well-received, though for distinct reasons. The PBL group's appreciation for the cultivation of thinking abilities and the CBL group's emphasis on the connection to real-world practice highlight the different learning experiences each method offers. For students who are academically inclined toward critical analysis, PBL may be more suitable, whereas those focused on immediate clinical application may prefer CBL (26).

Given the complementary strengths observed, we advocate for an Integrated (Hybrid) Teaching Model. Instead of choosing one over the other, educators should combine them organically based on the specific phase of the curriculum. Specifically, we propose that PBL-driven modules be utilized in the early stages of the course to establish a robust theoretical foundation and research skills. Subsequently, CBL-driven modules can be introduced during the pre-clinical and clinical internship phases to enhance practical application, decision-making, and clinical judgment. Such a tiered approach ensures that students develop both the “how” (clinical skills) and the “why” (theoretical reasoning) necessary for high-quality endodontic care. Beyond curriculum design, factors such as ‘teacher's teaching pressure' and the ‘students' adaptation process' play critical roles. PBL requires instructors to transition from lecturers to facilitators, which increases cognitive load during sessions. Similarly, students initially accustomed to passive learning may face an adaptation period where self-directed research feels overwhelming.

Despite the positive findings, this study has several limitations that should be acknowledged. Firstly, the sample size of 156 students from a single institution may limit the generalizability of the findings; therefore, future multi-center trials involving larger and more diverse cohorts are necessary. Furthermore, since the study was conducted over a single semester, long-term follow-up is required to assess the durability of these learning outcomes and their impact on actual clinical performance after graduation. Another notable limitation is the absence of a traditional lecture-based learning (LBL) control group. This was a deliberate choice aligned with our institution's curriculum reform, which prioritizes student-centered pedagogies. Future research could incorporate a baseline LBL group to further quantify the incremental benefits of PBL and CBL in the specific context of dental endodontics.

## 5 Conclusion

In conclusion, both PBL and CBL have their own unique merits in dental endodontics education. Instead of advocating for one method over the other, educators should consider integrating PBL and CBL to create a more comprehensive and effective learning experience. For example, PBL could be used at the beginning of a course to build a strong theoretical foundation and develop critical thinking skills, while CBL could be introduced later to enhance practical application and clinical judgment. By leveraging the strengths of both methods, educators can better prepare dental students for the challenges of their future clinical practice, ultimately improving the quality of dental care.

## Data Availability

The raw data supporting the conclusions of this article will be made available by the authors, without undue reservation.
