# Supplementary material for: Comparison of the teaching effect of problem-based learning and case-based learning teaching methods in dental endodontics education
Source: Front Med (Lausanne). 2026 May 12;13:1800657. doi: 10.3389/fmed.2026.1800657 (PMC13201110; doi:10.3389/fmed.2026.1800657)
Supplement: Supplementary file 1 [file Supplementary_file_1.docx]

**Satisfaction Assessment Of Teaching Model**

**I. Instructions**

Please rate your level of agreement with each statement on a 5 - point Likert scale, where 1 represents "Strongly Disagree", 2 represents "Disagree", 3 represents "Neutral", 4 represents "Agree", and 5 represents "Strongly Agree". Your responses will be used to evaluate the teaching model and help improve the teaching quality of dental endodontics.

**II. Questionnaire Content**

**1. Effectiveness of Teaching Methods**

**1. The teaching method in dental endodontics has effectively enhanced my understanding of complex endodontic concepts.**

- 1. Strongly Disagree

- 2. Disagree

- 3. Neutral

- 4. Agree

- 5. Strongly Agree

**2. I feel that the teaching approach has significantly improved my ability to analyze and solve endodontic problems.**

- 1. Strongly Disagree

- 2. Disagree

- 3. Neutral

- 4. Agree

- 5. Strongly Agree

**3. The teaching method has been successful in helping me connect different aspects of endodontics knowledge.**

- 1. Strongly Disagree

- 2. Disagree

- 3. Neutral

- 4. Agree

- 5. Strongly Agree

**4. I believe that the teaching method has prepared me well for future endodontic clinical practice.**

- 1. Strongly Disagree

- 2. Disagree

- 3. Neutral

- 4. Agree

- 5. Strongly Agree

**5. Overall, I think the teaching method used in dental endodontics is highly effective.**

- 1. Strongly Disagree

- 2. Disagree

- 3. Neutral

- 4. Agree

- 5. Strongly Agree

**2. Learning Effect Improvement**

**1. After this semester's study of dental endodontics, I have seen a clear improvement in my theoretical knowledge.**

- 1. Strongly Disagree

- 2. Disagree

- 3. Neutral

- 4. Agree

- 5. Strongly Agree

**2. My practical skills in endodontics, such as instrument operation and treatment techniques, have improved a lot.**

- 1. Strongly Disagree

- 2. Disagree

- 3. Neutral

- 4. Agree

- 5. Strongly Agree

**3. I am more confident in my ability to diagnose and treat endodontic diseases compared to before.**

- 1. Strongly Disagree

- 2. Disagree

- 3. Neutral

- 4. Agree

- 5. Strongly Agree

**4. The learning experience has enhanced my ability to apply endodontic knowledge in real - world scenarios.**

- 1. Strongly Disagree

- 2. Disagree

- 3. Neutral

- 4. Agree

- 5. Strongly Agree

**5. I think my overall learning performance in dental endodontics has improved significantly.**

- 1. Strongly Disagree

- 2. Disagree

- 3. Neutral

- 4. Agree

- 5. Strongly Agree

**3. Curriculum Rationality**

**1. The structure of the dental endodontics curriculum is logical and helps me build a solid knowledge system.**

- 1. Strongly Disagree

- 2. Disagree

- 3. Neutral

- 4. Agree

- 5. Strongly Agree

**2. The content arrangement of the curriculum is in line with the difficulty level and learning progress of dental endodontics.**

- 1. Strongly Disagree

- 2. Disagree

- 3. Neutral

- 4. Agree

- 5. Strongly Agree

**3. The cases and examples used in the curriculum are relevant and effective in explaining endodontic concepts.**

- 1. Strongly Disagree

- 2. Disagree

- 3. Neutral

- 4. Agree

- 5. Strongly Agree

**4. The curriculum provides sufficient opportunities for me to practice and apply what I have learned in endodontics.**

- 1. Strongly Disagree

- 2. Disagree

- 3. Neutral

- 4. Agree

- 5. Strongly Agree

**5. Overall, I think the dental endodontics curriculum is well - designed and rational.**

- 1. Strongly Disagree

- 2. Disagree

- 3. Neutral

- 4. Agree

- 5. Strongly Agree

**4. Faculty Guidance Role**

**1. My teachers' guidance in dental endodontics has been very helpful in clarifying my doubts.**

- 1. Strongly Disagree

- 2. Disagree

- 3. Neutral

- 4. Agree

- 5. Strongly Agree

**2. Teachers' feedback on my assignments and presentations has effectively improved my understanding of endodontics.**

- 1. Strongly Disagree

- 2. Disagree

- 3. Neutral

- 4. Agree

- 5. Strongly Agree

**3. My teachers can effectively guide me to think deeply about endodontic problems and find solutions.**

- 1. Strongly Disagree

- 2. Disagree

- 3. Neutral

- 4. Agree

- 5. Strongly Agree

**4. Teachers' explanations and demonstrations in class have made endodontic knowledge easier to understand.**

- 1. Strongly Disagree

- 2. Disagree

- 3. Neutral

- 4. Agree

- 5. Strongly Agree

**5. Overall, I am satisfied with the guidance and support provided by my teachers in dental endodontics.**

- 1. Strongly Disagree

- 2. Disagree

- 3. Neutral

- 4. Agree

- 5. Strongly Agree

**5. Learning Experience Feelings**

**1. I enjoy the process of learning dental endodontics and find it interesting.**

- 1. Strongly Disagree

- 2. Disagree

- 3. Neutral

- 4. Agree

- 5. Strongly Agree

**2. I feel a sense of achievement when I master a new endodontic concept or skill.**

- 1. Strongly Disagree

- 2. Disagree

- 3. Neutral

- 4. Agree

- 5. Strongly Agree

**3. The learning atmosphere in dental endodontics classes makes me more motivated to learn.**

- 1. Strongly Disagree

- 2. Disagree

- 3. Neutral

- 4. Agree

- 5. Strongly Agree

**4. I like the group activities in dental endodontics learning, which help me communicate and collaborate with my peers.**

- 1. Strongly Disagree

- 2. Disagree

- 3. Neutral

- 4. Agree

- 5. Strongly Agree

**5. Overall, I have a positive learning experience in dental endodontics.**

- 1. Strongly Disagree

- 2. Disagree

- 3. Neutral

- 4. Agree

- 5. Strongly Agree
