# Supplementary material for: Comparison of the teaching effect of problem-based learning and case-based learning teaching methods in dental endodontics education
Source: Front Med (Lausanne). 2026 May 12;13:1800657. doi: 10.3389/fmed.2026.1800657 (PMC13201110; doi:10.3389/fmed.2026.1800657)
Supplement: Supplementary file 2 [file Supplementary_file_2.docx]

**Self - learning Ability Assessment**

**I. Learning Plan And Management Ability (20 points)**

**1. When you start to study a complex endodontics topic like root canal treatment complications, how do you plan your study time?**

A. I make a detailed weekly schedule, allocating specific hours each day for reading textbooks, researching online, and discussing with classmates. (4 points)

B. I plan to study it over a few days, roughly dividing time for different learning resources but without a strict schedule. (3 points)

C. I just start studying when I have time, without a clear time - division plan. (2 points)

D. I only study when there is an upcoming test related to it, without any prior plan. (1 point)

E. I don't have a study plan for such topics at all. (0 points)

**2. If you set a goal to master the diagnosis of apical periodontitis in one week, how do you monitor your progress?**

A. I create a checklist of key knowledge points and mark them off as I learn, and I also test myself every day. (4 points)

B. I check if I can answer some simple questions about it every few days. (3 points)

C. I rely on my classmates to tell me if I seem to understand it. (2 points)

D. I don't really monitor my progress; I just keep studying until I feel like I know it. (1 point)

E. I don't think about monitoring my progress. (0 points)

**3. When you encounter difficulties in understanding the content of endodontics instrument usage in your study, how do you adjust your learning plan?**

A. I immediately adjust my schedule, allocate more time to this part, and seek additional resources like videos or professional forums. (4 points)

B. I try to find more examples in textbooks to help me understand, and spend a bit more time on it. (3 points)

C. I ask my classmates for help, but don't change my study plan much. (2 points)

D. I just skip it and hope to understand it later. (1 point)

E. I don't know how to adjust my plan. (0 points)

**4. How do you balance your study of endodontics with other dental courses?**

A. I make a comprehensive study plan for all dental courses, prioritizing tasks based on difficulty and importance, and allocate time proportionally. (4 points)

B. I study each course as they come, trying to give more time to difficult ones. (3 points)

C. I focus more on the courses I like, and spend less time on endodontics if I'm not interested. (2 points)

D. I just study randomly, without considering the balance. (1 point)

E. I don't care about the balance. (0 points)

**5. In a long - term endodontics project, how do you ensure that you complete each stage on time?**

A. I break the project into small tasks, set deadlines for each task, and regularly review my progress. (4 points)

B. I try to complete the project as a whole, but may not meet the deadlines exactly. (3 points)

C. I rely on my group members to remind me of the deadlines. (2 points)

D. I often delay the project until the last minute. (1 point)

E. I don't care about meeting the deadlines. (0 points)

**II. Information Acquisition And Screening Ability (20 points)**

**1. When researching about the latest endodontics filling materials, which resources do you use first?**

A. I first search in academic databases like PubMed and CNKI, then refer to dental professional websites. (4 points)

B. I start with dental textbooks, and then look for information on the Internet. (3 points)

C. I search on general search engines like Google. (2 points)

D. I ask my classmates for information. (1 point)

E. I don't know where to start looking for information. (0 points)

**2. How do you determine if the information about endodontics treatment techniques from a website is reliable?**

A. I check the source of the information, such as the author's credentials, and look for citations from reliable studies. (4 points)

B. I see if the website looks professional. (3 points)

C. I compare it with what I know from textbooks. (2 points)

D. I just believe it if it seems reasonable. (1 point)

E. I don't know how to judge the reliability. (0 points)

**3. You found two different research papers about the same endodontics topic, but they have conflicting results. What do you do?**

A. I carefully analyze the research methods, sample sizes, and limitations of both papers, and then look for more relevant studies. (4 points)

B. I choose to believe the paper from a more well - known journal. (3 points)

C. I ask my teacher which one is correct. (2 points)

D. I'm confused and don't know what to do. (1 point)

E. I ignore the conflict and use the information randomly. (0 points)

**4. When using an academic database to search for endodontics literature, how do you refine your search results?**

A. I use specific keywords, limit the publication date, and filter by the type of study (e.g., clinical trial, review). (4 points)

B. I just use general keywords and browse through the results. (3 points)

C. I ask someone else to help me refine the search. (2 points)

D. I don't know how to refine the search. (1 point)

E. I don't care about refining the search results. (0 points)

**5. In the process of gathering information on endodontics, if you find a lot of irrelevant information, what will you do?**

A. I use advanced search techniques to exclude irrelevant terms, and also carefully screen the information by quickly skimming through the abstracts. (4 points)

B. I try to read through all the information to find what I need. (3 points)

C. I ask my classmates to help me sort out the information. (2 points)

D. I give up and start over. (1 point)

E. I don't know how to deal with it. (0 points)

**III. Knowledge Understanding And Transformation Ability (20 points)**

**1. After learning about the normal anatomy of the dental pulp, how would you explain the changes in pulp anatomy in a case of chronic pulpitis?**

A. I can clearly explain the pathological changes in pulp cells, blood vessels, and nerves based on my knowledge of normal anatomy, and relate it to the symptoms of chronic pulpitis. (4 points)

B. I can mention some changes in pulp, but may not be able to fully connect them with the symptoms. (3 points)

C. I only know the normal pulp anatomy, and can't say much about the changes in chronic pulpitis. (2 points)

D. I'm not sure what the changes are. (1 point)

E. I don't know anything about this. (0 points)

**2. Given a new type of endodontics treatment device, how do you apply your existing knowledge to understand its working principle?**

A. I can analyze its structure, compare it with known treatment devices, and use my knowledge of endodontics treatment concepts to figure out its working principle. (4 points)

B. I can make some guesses based on its appearance, but may not be very accurate. (3 points)

C. I need to read the instructions to understand it. (2 points)

D. I have no idea how to understand its working principle. (1 point)

E. I don't care about understanding its working principle. (0 points)

**3. You learned about the principle of root canal disinfection. How would you apply this knowledge to design a new disinfection protocol?**

A. I can combine different disinfection methods based on the principle, considering factors like the type of infection, and design a comprehensive disinfection protocol. (4 points)

B. I can suggest some common disinfection methods, but may not be able to design a new protocol. (3 points)

C. I only know the existing disinfection methods, and can't design a new one. (2 points)

D. I don't know how to start designing. (1 point)

E. I don't think I can design a new protocol. (0 points)

**4. If you encounter a complex endodontics case in clinical practice that is different from what you learned in class, how do you use your knowledge to deal with it?**

A. I can analyze the case from multiple aspects, integrate relevant knowledge, and come up with a possible treatment plan. (4 points)

B. I can recall some similar cases in class and try to apply the same treatment method. (3 points)

C. I'm not sure what to do and will wait for my supervisor's advice. (2 points)

D. I'm completely lost and don't know how to use my knowledge. (1 point)

E. I don't believe my knowledge can help in this situation. (0 points)

**5. After learning about the different types of endodontics filling materials, how would you choose the most suitable one for a specific case in a real - world scenario?**

A. I can consider factors like the patient's condition, the type of tooth, and the potential for future treatment, and make a well - informed choice. (4 points)

B. I can choose based on some common factors, but may miss some important considerations. (3 points)

C. I just choose the most commonly used material. (2 points)

D. I'm not sure how to choose the right material. (1 point)

E. I don't know the differences between the filling materials. (0 points)

**IV. Problem Solving And Reflection Ability (20 points)**

**1. During a root canal treatment simulation, you find that the root canal is severely curved and difficult to instrument. What do you do?**

A. I consider different techniques such as using flexible files, changing the filing angle, and reflect on the potential risks and benefits of each approach. (4 points)

B. I try one or two common methods and hope it works. (3 points)

C. I ask my partner or teacher for help immediately. (2 points)

D. I'm stuck and don't know what to do. (1 point)

E. I give up and stop the simulation. (0 points)

**2. After presenting your group's endodontics case analysis in class, and receiving some negative feedback from classmates and teachers, what do you do?**

A. I carefully analyze the feedback, identify the areas for improvement, and think about how to avoid similar mistakes in the future. (4 points)

B. I listen to the feedback, but may not take it seriously. (3 points)

C. I feel bad, but don't really think about how to improve. (2 points)

D. I ignore the feedback. (1 point)

E. I get angry and don't want to participate in such activities anymore. (0 points)

**3. You are working on an endodontics research project, but the results you get are not as expected. What is your approach?**

A. I review the research process, check for possible errors in methods or data collection, and consider alternative explanations for the results. (4 points)

B. I assume it's just a coincidence and continue with the project. (3 points)

C. I ask my supervisor what to do. (2 points)

D. I'm confused and don't know how to proceed. (1 point)

E. I give up on the project. (0 points)

**4. In a group endodontics study, there is a conflict among group members about the diagnosis of a case. How do you solve it?**

A. I encourage everyone to present their reasoning, analyze the evidence together, and try to reach a consensus through discussion. (4 points)

B. I try to persuade others to agree with my opinion. (3 points)

C. I let the group leader make the decision. (2 points)

D. I don't participate in the discussion and let them solve it. (1 point)

E. I make the situation worse by arguing. (0 points)

**5. If you make a mistake in a practical endodontics operation, such as over - filing the root canal, what do you do?**

A. I immediately stop, assess the situation, think about the possible consequences, and learn from this mistake to avoid repeating it in the future. (4 points)

B. I try to fix it as best as I can, but don't think much about why it happened. (3 points)

C. I tell my teacher and wait for them to handle it. (2 points)

D. I'm afraid and don't know what to do. (1 point)

E. I pretend nothing happened. (0 points)

**V. Learning Method Innovation And Application Ability (20 points)**

**1. To remember the complex steps of endodontics treatment, what new learning method do you try?**

A. I create mind maps to organize the steps, and use mnemonic devices to remember key points. (4 points)

B. I make flashcards to review the steps. (3 points)

C. I just repeat the steps many times. (2 points)

D. I don't try any new method. (1 point)

E. I don't care about remembering the steps. (0 points)

**2. In the study of endodontics diseases, how do you apply technology to enhance your learning?**

A. I use virtual reality or 3D - modeling software to visualize the diseases and their treatment processes. (4 points)

B. I watch educational videos on platforms like YouTube. (3 points)

C. I just use my mobile phone to look up some text information. (2 points)

D. I don't use technology to learn. (1 point)

E. I don't know how technology can help in learning endodontics. (0 points)

**3. When studying a difficult endodontics concept, such as the mechanism of pulp inflammation, what innovative way do you use to understand it?**

A. I create a short story or a metaphor to represent the concept, making it easier to understand. (4 points)

B. I try to find more examples in real life to help me understand. (3 points)

C. I read more textbooks about it. (2 points)

D. I don't know how to understand it better. (1 point)

E. I give up on understanding this concept. (0 points)

**4. How do you apply group - learning in endodontics to innovate your learning experience?**

A. I organize group discussions in a new way, such as role - playing different parts in a treatment process, to deepen understanding. (4 points)

B. I just participate in normal group discussions. (3 points)

C. I don't like group - learning and prefer to study alone. (2 points)

D. I don't know how to make group - learning more innovative. (1 point)

E. I avoid group - learning. (0 points)

**5. To better understand the relationship between different endodontics topics, what new learning strategy do you adopt?**

A. I create a concept map to show the connections between topics, and use color - coding to distinguish different relationships. (4 points)

B. I list the topics side by side to see the differences. (3 points)

C. I don't think about the relationships between topics. (2 points)

D. I don't know how to show the relationships. (1 point)

E. I don't care about the relationships between topics. (0 points)
