# Supplementary material for: Comparison of the teaching effect of problem-based learning and case-based learning teaching methods in dental endodontics education
Source: Front Med (Lausanne). 2026 May 12;13:1800657. doi: 10.3389/fmed.2026.1800657 (PMC13201110; doi:10.3389/fmed.2026.1800657)
Supplement: Supplementary file 3 [file Supplementary_file_3.docx]

**Practical Skill Assessment in Dental Endodontics**

**I. Instrument Operation Specification (20 points)**

**A. Root Canal File Usage (10 points)**

**1. Insertion Angle (3 points)**

- Correct insertion angle within the recommended range (±5° of the ideal angle for the specific tooth type) during root canal preparation: 3 points.

- Deviation of 5 - 10°: 2 points.

- Deviation greater than 10°: 0 - 1 point.

**2. Filing Pressure (4 points)**

- Maintains proper filing pressure, avoiding excessive force that could cause root canal perforation or apical transportation, and insufficient force that may lead to ineffective canal shaping: 4 points.

- Shows minor fluctuations in pressure but still manages to perform the task: 2 - 3 points.

- Repeatedly applies inappropriate pressure, affecting the quality of canal preparation: 0 - 1 point.

**3. File Rotation and Manipulation (3 points)**

- Demonstrates smooth and coordinated file rotation, following the correct technique (e.g., balanced force technique or crown - down technique as appropriate): 3 points.

- Some hesitation or unsmoothness in rotation, but can still complete the basic operation: 2 points.

- Erratic rotation or incorrect manipulation that may damage the file or the root canal: 0 - 1 point.

**B. Usage of Other Instruments (e.g., Endodontic Burs for Access Cavity Preparation) (10 points)**

**1. Selection of the Right Burs (3 points)**

- Selects the appropriate endodontic burs based on the tooth type, location of the lesion, and the purpose of access cavity preparation: 3 points.

- Chooses a sub - optimal but still usable burs: 2 points.

- Selects an incorrect or inappropriate burs: 0 - 1 point.

**2. Control of the Handpiece (4 points)**

- Maintains stable and controlled handpiece operation, with proper speed and pressure adjustment during access cavity preparation: 4 points.

- Minor instability in handpiece control, but does not significantly affect the quality of the access cavity: 2 - 3 points.

- Uncontrolled handpiece movement, resulting in an irregular or incorrect access cavity: 0 - 1 point.

**3. Achievement of the Ideal Access Cavity Shape (3 points)**

- Creates an access cavity with the correct shape, size, and location, providing unobstructed access to the root canal system: 3 points.

- The access cavity has minor flaws but still allows for root canal treatment: 2 points.

- The access cavity is severely defective, making root canal treatment difficult or impossible: 0 - 1 point.

**II. Therapeutic Technical Proficiency (20 points)**

**A. Root Canal Treatment Procedures (10 points)**

**1. Pulp Chamber Access (3 points)**

- Gains complete and unobstructed access to the pulp chamber quickly and accurately, without causing unnecessary damage to the tooth structure: 3 points.

- Takes a bit longer or causes minor damage but still manages to access the pulp chamber: 2 points.

- Has difficulty accessing the pulp chamber or causes significant damage: 0 - 1 point.

**2. Root Canal Cleaning and Shaping (4 points)**

- Cleans the root canal thoroughly, removing all pulp tissue, debris, and bacteria, and shapes the canal to the appropriate taper and size: 4 points.

- Some residue remains in the canal or the shaping is not perfect but still meets the basic requirements: 2 - 3 points.

- Incomplete cleaning or improper shaping that may affect the treatment outcome: 0 - 1 point.

**3. Root Canal Obturation (3 points)**

- Performs root canal obturation with proper filling material, achieving a complete and dense seal, without over - filling or under - filling: 3 points.

- Slight over - filling or under - filling, but not likely to cause major problems: 2 points.

- Significant over - filling or under - filling that may lead to treatment failure: 0 - 1 point.

**B. Management of Complications during Treatment (10 points)**

**1. Recognition of Complications (4 points)**

- Can quickly and accurately recognize potential complications during root canal treatment, such as root canal perforation, instrument separation, or apical extrusion of debris: 4 points.

- Recognizes the complication after a short delay but still in time to take appropriate measures: 2 - 3 points.

- Fails to recognize the complication or realizes it too late: 0 - 1 point.

**2. Response to Complications (6 points)**

- Takes immediate and appropriate measures to deal with the complication, minimizing its impact on the treatment outcome and the patient's condition: 6 points.

- Tries to address the complication but the measures are not fully effective: 3 - 5 points.

- Does not know how to respond or takes inappropriate actions: 0 - 2 points.

**III. Material Selection Rationality (20 points)**

**A. Selection of Root Canal Filling Materials (10 points)**

**1. Consideration of Tooth and Patient Factors (5 points)**

- Selects the root canal filling material based on factors such as the type of tooth (anterior or posterior), the patient's overall health (e.g., presence of systemic diseases), and the condition of the root canal (e.g., shape, length, presence of infection): 5 points.

- Takes some but not all relevant factors into account: 3 - 4 points.

- Ignores important factors when choosing the filling material: 0 - 2 points.

**2. Knowledge of Material Properties (5 points)**

- Demonstrates a good understanding of the properties of different root canal filling materials (e.g., setting time, biocompatibility, sealing ability), and selects the material that best suits the case: 5 points.

- Has a basic understanding but makes some minor mistakes in material selection: 3 - 4 points.

- Lacks knowledge of material properties and makes inappropriate choices: 0 - 2 points.

**B. Selection of Other Endodontic Materials (e.g., Irrigants, Medicaments) (10 points)**

**1. Appropriate Selection for the Case (5 points)**

- Selects the correct irrigants and medicaments according to the stage of treatment, the type of infection, and the patient's symptoms. For example, choosing an antibacterial irrigant for an infected root canal: 5 points.

- Makes a sub - optimal but still acceptable choice: 3 - 4 points.

- Selects an inappropriate irrigant or medicament: 0 - 2 points.

**2. Understanding of Material Functions (5 points)**

- Can clearly explain the functions of the selected irrigants and medicaments, and how they contribute to the success of the endodontic treatment: 5 points.

- Has a general idea but may miss some key functions: 3 - 4 points.

- Does not understand the functions of the materials: 0 - 2 points.

**IV. Aseptic Operation Compliance (20 points)**

**A. Pre - treatment Asepsis (6 points)**

**1. Sterilization of Instruments (3 points)**

- Ensures that all endodontic instruments are properly sterilized before use, following the correct sterilization procedures (e.g., autoclaving at the appropriate temperature and time): 3 points.

- There are minor issues with instrument sterilization, such as improper packaging or a slightly shorter sterilization time, but still within an acceptable range: 2 points.

- Fails to sterilize the instruments properly: 0 - 1 point.

**2. Operator Hygiene (3 points)**

- The operator wears appropriate personal protective equipment (PPE), including gloves, mask, and protective eyewear, and follows proper hand - washing procedures before starting the treatment: 3 points.

- Some parts of PPE are missing or hand - washing is not thorough: 2 points.

- Does not comply with basic operator hygiene requirements: 0 - 1 point.

**B. Intra - treatment Asepsis (8 points)**

**1. Rubber Dam Isolation (4 points)**

- Applies the rubber dam correctly, achieving a complete and effective isolation of the treated tooth, preventing contamination from the oral cavity: 4 points.

- The rubber dam is not properly applied, but still provides partial isolation: 2 - 3 points.

- Fails to use the rubber dam or uses it ineffectively: 0 - 1 point.

**2. Handling of Instruments and Materials during Treatment (4 points)**

- Keeps instruments and materials sterile during the treatment process. For example, does not touch the sterile parts of the instruments with non - sterile hands and stores materials in a clean and covered container: 4 points.

- Has some minor breaches of asepsis during instrument and material handling: 2 - 3 points.

- Repeatedly violates aseptic rules during the treatment: 0 - 1 point.

**C. Post - treatment Asepsis (6 points)**

**1. Disposal of Instruments and Waste (3 points)**

- Disposes of used endodontic instruments and waste in a proper manner, following the relevant regulations for medical waste disposal: 3 points.

- Improper disposal but still within an acceptable range: 2 points.

- Fails to dispose of waste and instruments properly: 0 - 1 point.

**2. Cleaning and Disinfection of the Work Area (3 points)**

- Cleans and disinfects the work area thoroughly after the treatment, removing all debris and contaminants: 3 points.

- Performs an incomplete cleaning or disinfection: 2 points.

- Does not clean or disinfect the work area at all: 0 - 1 point.

**V. Accuracy of Clinical Judgment (20 points)**

**A. Diagnosis Based on Clinical and Radiographic Findings (10 points)**

**1. Interpretation of Clinical Symptoms (4 points)**

- Correctly interprets the patient's symptoms (e.g., pain characteristics, sensitivity to stimuli) and uses them to narrow down the possible diagnoses: 4 points.

- Misinterprets some symptoms or fails to consider all relevant symptoms: 2 - 3 points.

- Seriously misinterprets the symptoms, leading to an incorrect diagnosis: 0 - 1 point.

**2. Analysis of Radiographic Images (6 points)**

- Analyzes the radiographic images accurately, identifying the presence of carious lesions, periapical radiolucencies, root canal anatomy, and other relevant features. Can also distinguish normal from abnormal radiographic findings: 6 points.

- Misses some important radiographic features or makes minor misinterpretations: 3 - 5 points.

- Seriously misinterprets the radiographic images, resulting in an incorrect diagnosis: 0 - 2 points.

**B. Treatment Planning Based on the Diagnosis (10 points)**

**1. Formulation of a Comprehensive Treatment Plan (5 points)**

- Develops a comprehensive and appropriate treatment plan based on the diagnosis, considering the patient's overall oral health, the condition of the tooth, and the patient's preferences: 5 points.

- The treatment plan has some minor flaws or does not fully consider all relevant factors: 3 - 4 points.

- The treatment plan is inappropriate or incomplete: 0 - 2 points.

**2. Justification of the Treatment Plan (5 points)**

- Can clearly justify each step of the treatment plan, explaining the rationale behind the chosen treatment methods, the expected outcomes, and the potential risks: 5 points.

- Can provide a basic justification but may miss some important points: 3 - 4 points.

- Cannot justify the treatment plan or provides incorrect justifications: 0 - 2 points.
