# Supplementary material for: Comparison of the teaching effect of problem-based learning and case-based learning teaching methods in dental endodontics education
Source: Front Med (Lausanne). 2026 May 12;13:1800657. doi: 10.3389/fmed.2026.1800657 (PMC13201110; doi:10.3389/fmed.2026.1800657)
Supplement: Supplementary file 4 [file Supplementary_file_4.docx]

**Dental Endodontics Theoretical Knowledge Test**

**I. Multiple - Choice Questions (50 points, 2 points per question)**

1. Which of the following is the main function of the dental pulp?

A. Providing nutrition to the dentin

B. Protecting the tooth from external forces

C. Aiding in chewing

D. Assisting in tooth eruption

2. What is the most common cause of pulpitis?

A. Trauma

B. Bacterial infection

C. Chemical irritation

D. Thermal injury

3. Which diagnostic tool is most useful for detecting early apical periodontitis?

A. Visual inspection

B. Percussion test

C. Radiograph

D. Palpation

4. In root canal preparation, the main purpose of using dental files is to:

A. Remove the pulp tissue

B. Shape the root canal

C. Disinfect the root canal

D. Seal the root canal

5. Which of the following materials is commonly used for root canal filling?

A. Amalgam

B. Composite resin

C. Gutta - percha

D. Zinc oxide eugenol

6. What is the role of calcium hydroxide in endodontic treatment?

A. It is a filling material

B. It has antibacterial and pulp - promoting - healing properties

C. It is used for temporary restoration

D. It helps in tooth whitening

7. The dental pulp is rich in:

A. Blood vessels only

B. Nerves only

C. Both blood vessels and nerves

D. Lymphatic vessels only

8. Which of the following symptoms is typical of irreversible pulpitis?

A. Mild pain when biting

B. Spontaneous, severe pain

C. Slight sensitivity to cold

D. Pain relieved by hot food

9. When performing a pulp vitality test, what does a negative response usually indicate?

A. Normal pulp vitality

B. Reversible pulpitis

C. Irreversible pulpitis

D. Necrotic pulp

10. The ideal length for root canal filling is:

A. Exactly to the root apex

B. 1 - 2 mm short of the root apex

C. 2 - 3 mm beyond the root apex

D. It doesn't matter as long as the canal is filled

11. Which bacteria are commonly associated with apical periodontitis?

A. Streptococcus mutans

B. Lactobacillus

C. Porphyromonas gingivalis

D. Enterococcus faecalis

12. What is the main difference between direct pulp capping and indirect pulp capping?

A. The location of the capping material

B. The type of capping material used

C. Whether the pulp is exposed or not

D. The prognosis of the treatment

13. In endodontic treatment, the concept of "working length" refers to:

A. The length of the dental file used

B. The distance from the incisal edge to the root apex

C. The distance from the reference point to the apical constriction

D. The length of the root canal

14. Which of the following factors can affect the success rate of endodontic treatment?

A. The skill of the operator

B. The type of bacteria in the root canal

C. The quality of the filling material

D. All of the above

15. What is the purpose of using a rubber dam during endodontic treatment?

A. To isolate the tooth

B. To protect the patient's airway

C. To improve visibility

D. All of the above

16. Which of the following is a contraindication for endodontic treatment?

A. A large carious lesion

B. Severe periodontal disease with hopeless prognosis

C. Mild tooth mobility

D. Pulpitis

17. When diagnosing pulpitis, which symptom is more reliable for differentiating between reversible and irreversible pulpitis?

A. Duration of pain

B. Intensity of pain

C. Response to thermal stimuli

D. All of the above

18. The first step in endodontic treatment is usually:

A. Root canal preparation

B. Pulp extirpation

C. Access cavity preparation

D. Root canal filling

19. Which of the following is not a part of the periapical tissues?

A. Alveolar bone

B. Periodontal ligament

C. Cementum

D. Enamel

20. In a case of a tooth with a deep carious lesion close to the pulp, but no pulp exposure, what treatment might be considered first?

A. Root canal treatment

B. Indirect pulp capping

C. Direct pulp capping

D. Extraction

21. The main function of the periapical tissues is to:

A. Support and protect the tooth

B. Provide nutrition to the pulp

C. Aid in tooth eruption

D. Participate in tooth movement

22. What is the most common complication during root canal preparation?

A. Root canal perforation

B. Over - instrumentation

C. Instrument separation

D. All of the above

23. Which of the following is an advantage of using rotary nickel - titanium files in root canal preparation?

A. They are more flexible

B. They can shape the root canal faster

C. They have a lower risk of instrument separation

D. All of the above

24. In endodontic diagnosis, the electric pulp tester measures:

A. The electrical conductivity of the pulp

B. The blood flow in the pulp

C. The nerve sensitivity of the pulp

D. The temperature of the pulp

25. When treating a tooth with apical periodontitis, the main goal is to:

A. Eliminate the infection in the root canal

B. Promote the healing of the periapical tissues

C. Both A and B

D. Restore the tooth's appearance

**II. Short - Answer Questions (50 points, 10 points per question)**

1. Briefly describe the anatomical structure of the dental pulp and explain how its structure relates to its functions.

2. Explain the pathogenesis of apical periodontitis, including the role of bacteria, the immune response, and the changes in the periapical tissues.

3. Discuss the importance of accurate diagnosis in endodontics. List at least three diagnostic methods and explain how they are used in combination to make a reliable diagnosis of pulpitis or apical periodontitis.

4. Describe the process of root canal preparation in detail, including the steps involved, the instruments used, and the key considerations for successful preparation.

5. Compare and contrast direct pulp capping and pulpotomy. Include the indications, treatment procedures, and potential outcomes of each method.

**III. Case - Application Problems (50 points, 25 points per question)**

**Case 1**

A 35 - year - old patient presents with a chief complaint of severe, spontaneous pain in the upper right first molar for the past 3 days. The pain is aggravated by hot food and cold stimuli, and it wakes the patient up at night. Clinical examination reveals a large carious lesion on the occlusal surface of the tooth. There is no obvious swelling in the facial area. Percussion test of the tooth elicits mild pain. Radiograph shows a deep carious lesion approaching the pulp chamber, but no obvious periapical radiolucency.

1. What is your most likely diagnosis? Justify your answer based on the patient's symptoms and examination findings. (10 points)

2. Outline your proposed treatment plan. Include the specific procedures, the sequence of treatment, and the possible complications that you need to be aware of during the treatment process. (15 points)

**Case 2**

A 45 - year - old patient comes to your clinic with a toothache in the lower left second molar. The patient reports that the tooth has been sensitive to cold for a long time, and recently, the pain has become more severe and persistent. Clinical examination shows a large restoration on the tooth. The tooth is slightly mobile. Percussion test is positive, and palpation of the periapical area is tender. Radiograph shows a periapical radiolucency around the root apex of the tooth.

1. Analyze the possible causes of the patient's symptoms and the radiographic findings. (10 points)

2. Develop a comprehensive treatment plan for this patient. Consider the patient's overall oral health, the condition of the tooth, and the potential long - term prognosis. Explain the rationale behind each step of your treatment plan. (15 points)
